# The core role of central nervous system in sepsis-related organ damage

**DOI:** 10.3389/fimmu.2025.1694003

**Published:** 2025-11-21

**Authors:** Fajuan Tang, ShanShan Wu, Zhuan Zou, Xihong Li, Lina Qiao

**Affiliations:** 1 Department of Emergency, West China Second University Hospital, Sichuan University, Chengdu, China; 2 Key Laboratory of Birth Defects and Related Diseases of Women and Children, Sichuan University, Ministry of Education, Chengdu, China; 3 Department of Pediatrics, West China Second University Hospital, Sichuan University, Chengdu, China

**Keywords:** sepsis, neuroimmune regulation, autonomic nerve function, inflammation, sepsis-associated encephalopathy

## Abstract

Sepsis-induced multiple organ dysfunction syndrome is the leading cause of mortality among patients with sepsis. Its pathophysiological mechanisms encompass various factors, including dysregulated inflammatory responses, endothelial injury and microcirculatory disturbances, abnormal activation of cell death pathways, as well as metabolic reprogramming and immune interactions. The central nervous system (CNS) is one of the earliest and most susceptible organs affected during the septic process. This involvement not only results in brain dysfunction due to neuronal damage, excessive activation of microglia, and neuroinflammatory responses but also contributes to systemic organ damage through diverse neural regulatory mechanisms. Specifically, the CNS influences the function of distant organs via the autonomic nervous system—comprising inhibition of the vagus nerve cholinergic anti-inflammatory pathway and excessive activation of sympathetic nerve pathways—the neuroimmune regulatory network, central trained immunity regulation, extravasation of brain-derived inflammatory factors, and exosome transport. This paper provides a systematic review of key pathogenic mechanisms underlying sepsis-related organ damage while emphasizing the pivotal regulatory role played by the central nervous system in this pathological process along with its potential therapeutic implications.

## 1 Introduction

Sepsis is a life-threatening syndrome characterized by organ dysfunction resulting from a dysregulated host response to infection (1). This condition can lead to multi-organ system damage, primarily affecting vital organs such as the brain, heart, lungs, liver, and kidneys (2, 3). However, current clinical practice for treating sepsis-related organ damage predominantly relies on antibiotics and supportive care, with a notable absence of specific targeted therapeutic agents. Importantly, the central nervous system (CNS), recognized as one of the earliest and most vulnerable target organs (4), plays a critical role in the pathophysiology of sepsis. Clinical studies indicate that up to 70% of patients with sepsis develop sepsis-associated encephalopathy (SAE), which is characterized by diffuse brain dysfunction without direct evidence of central nervous system infection (5). his acute brain injury not only elevates the acute mortality rate among sepsis patients (ranging from 9% to 76%) (6) but also contributes to long-term cognitive impairment that severely impacts patients’ quality of life (7). Moreover, recent research has revealed that the CNS serves as a key driver in both the onset and progression of peripheral organ dysfunction and its long-term sequelae by modulating autonomic neural pathways and neuroinflammatory responses (8, 9). An experimental study have demonstrated that protective interventions targeting the nervous system—such as cholinergic pathway-targeted therapies—can effectively reverse multi-organ damage (10). These significant findings underscore the CNS’s central regulatory role in sepsis-related multi-organ dysfunction; however, elucidation of its regulatory mechanisms remains elusive. Therefore, an in-depth investigation into how the CNS influences sepsis-related organ damage will provide essential theoretical foundations and therapeutic targets for developing novel intervention strategies aimed at mitigating multi-organ damage associated with sepsis.

## 2 The core mechanism of sepsis-induced organ damage

**Figure 1:**
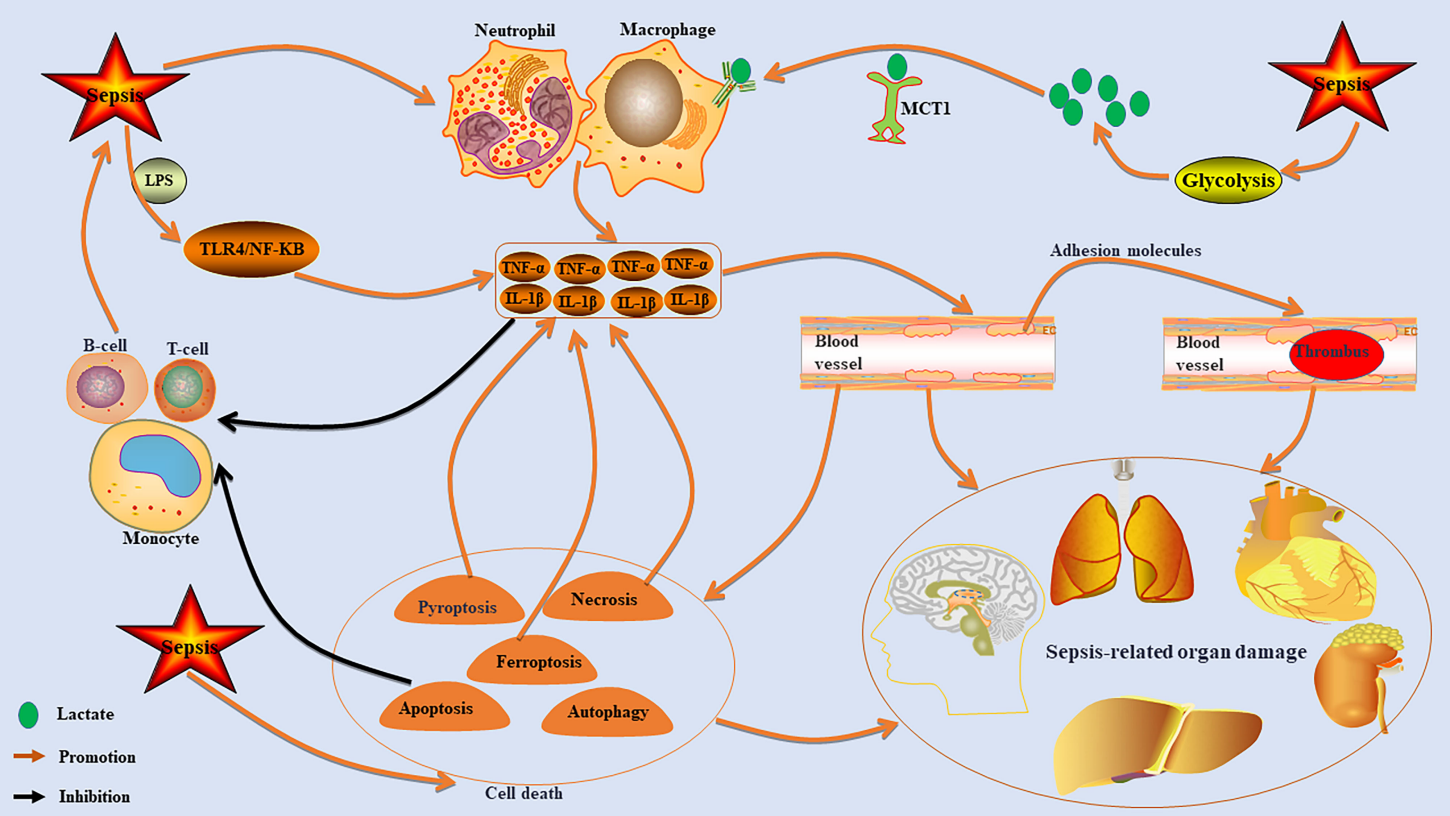
The core mechanism of sepsis-induced organ damage. Sepsis can lead to excessive activation of immune cells, such as macrophages and neutrophils, resulting in the release of high levels of pro-inflammatory factors like TNF-α and IL-1β. This cytokine storm damages endothelial cells, increases adhesion molecules, promotes microcirculation thrombosis, and causes ischemia and hypoxia, which can trigger multiple organ dysfunction. The inflammatory response also inhibits monocyte, T cell, and B cell functions, leading to immunosuppression that exacerbates sepsis. Additionally, sepsis activates cell death pathways that further increase inflammatory cytokine production. It also stimulates the glycolytic pathway to enhance lactate production, promoting macrophage activation via MCT1 transport.

The fundamental mechanism underlying sepsis-induced multiple organ damage arises from the dysregulated inflammatory response of the host to infection, which initiates a cascade of interrelated pathophysiological processes (**Figure 1**). The resultant excessive cytokine storm leads to endothelial cell injury and aberrant activation, subsequently promoting an overactivation of the coagulation system (11, 12). This series of events culminates in microcirculatory thrombosis, tissue ischemia and hypoxia, as well as cellular apoptosis, thereby resulting in multiple organ dysfunction (13). This core process is frequently accompanied by an immunosuppressive state and metabolic reprogramming (14–16), both of which further intensify disease progression. Such alterations increase the risk of immune paralysis and secondary infections, ultimately exacerbating patient prognosis.

### 2.1 Uncontrolled inflammatory response and immune suppression

Sepsis is characterized by an exaggerated inflammatory response in its early stages (17). The infection by pathogens activates immune cells, such as macrophages and neutrophils, prompting them to release substantial quantities of pro-inflammatory mediators, including tumor necrosis factor-α (TNF-α) and lnterleukin-1β (IL-1β), which in turn initiates a systemic inflammatory response (17). Among these immune cells, the abnormal activation of macrophages—particularly through M1 polarization—plays a pivotal role in driving the inflammatory cascade. The inflammatory mediators released can directly inflict damage on endothelial cells and parenchymal organs (18, 19). Furthermore, pathogen-associated molecular patterns, such as lipopolysaccharides (LPS), further intensify this uncontrolled systemic inflammatory response by continuously activating signaling pathways associated with inflammation, notably TLR4/NF-κB (20, 21). Significantly, the body may concurrently enter or swiftly transition into an immune-suppressed state marked by lymphocyte depletion and monocyte dysfunction (22). This condition elevates the risk for secondary infections. In summary, heightened inflammation and immune suppression frequently coexist within sepsis patients, creating an “immune paradox” that contributes to multiple organ dysfunction.

### 2.2 Endothelial injury and microcirculation disorder

Under septic conditions, the heparan sulfate glycocalyx structure on the surface of endothelial cells is compromised, leading to impaired vascular barrier function and an abnormal increase in permeability (23, 24). Consequently, this results in tissue edema and insufficient microcirculatory perfusion, ultimately causing ischemic injury to various organs (23, 24). Simultaneously, endothelial cell apoptosis intensifies, accompanied by a marked upregulation of adhesion molecules such as ICAM-1 and VCAM-1 (25, 26). This promotes aberrant leukocyte-endothelial cell adhesion and microthrombosis (25, 26). These pathological alterations further exacerbate microcirculatory disorders, creating a positive feedback loop of tissue hypoxia-inflammation-coagulation that ultimately leads to ischemic hypoxic injury across multiple organs.

### 2.3 Activation of cell death pathways

Apoptosis in sepsis exhibits a cell type-specific activation pattern. The excessive apoptosis of immune cells, such as dendritic cells, results in a reduction of their numbers and functional impairments, thereby promoting an immunosuppressive state that heightens the risk of secondary infections (16). Conversely, the inhibition of neutrophil apoptosis may lead to alternative death pathways, including necroptosis or pyroptosis, which can further aggravate organ damage (27). Necroptosis plays a pivotal role during both the early and progressive stages of sepsis by mediating the release of substantial quantities of damage-associated molecular patterns (28). This process continuously amplifies the inflammatory cascade and is closely associated with sepsis-related organ dysfunction (28). Furthermore, excessive activation of pyroptosis—driven by Gasdermin D-induced membrane perforation and subsequent release of inflammatory mediators such as IL-1β—exacerbates oxidative stress, endothelial dysfunction, and thrombosis, particularly in cases involving septic cardiomyopathy (29, 30). For instance, the activation of the NLRP3 inflammasome and subsequent caspase-1 activation lead to the release of IL-1β and pyroptosis, which can directly result in myocardial injury and lesions in other organs (31, 32). Ferroptosis, characterized as a form of iron-dependent lipid peroxidation-driven cell death, is significantly implicated in the pathological processes associated with sepsis-induced brain injury, cardiomyopathy, acute kidney injury, lung damage, and liver dysfunction (1, 33). This type of cell death can aggravate the inflammatory cascade during sepsis by promoting the release of inflammatory mediators and contributing to multi-organ damage (34). Notably, ferroptosis inhibitors exhibit organ-protective effects (35). Furthermore, dysregulation of autophagy within endothelial cells and parenchymal cells—such as obstruction of autophagic flux—can intensify cellular damage and is closely linked to both the onset and progression of sepsis-related organ dysfunction (31). In all, sepsis has the capacity to activate multiple pathways leading to programmed cell death; this not only results in direct cellular demise but also exacerbates multi-organ dysfunction through mechanisms such as amplification of inflammatory cascades and disruption of immune homeostasis. However, further exploration is required to elucidate the inter-regulatory network relationships among these pathways.

### 2.4 Metabolic-immune interactions

Metabolic disorders serve as a critical molecular foundation for the onset of sepsis and subsequent organ dysfunction (36). Throughout the progression of sepsis, host cells undergo metabolic reprogramming, which is primarily characterized by a shift in energy metabolism from oxidative phosphorylation to glycolysis (37). This metabolic transition is essential for the activation and pro-inflammatory function of immune cells, particularly macrophages (38). Enhanced glycolysis during the acute phase not only supports the pro-inflammatory response necessary to combat infection (39), but also partially mitigates the “energy crisis” induced by sepsis (37). Persistent metabolic reprogramming fosters a transformation in immune status from pro-inflammatory to immunosuppressive, thus increasing susceptibility to secondary infections (40). The metabolites derived from glycolysis exhibit dual roles: they serve as energy substrates while also acting as crucial regulatory signaling molecules within the immune system (41). For instance, lactate—a product of metabolism—can influence immune cell functionality through specific transporters (such as MCT1 and MCT4) and receptors like GPR81 (42). Distinct patterns of immune dysfunction are associated with specific metabolic disorder profiles (43, 44). The immunosuppressive state observed in sepsis is accompanied by significant metabolic disturbances (45, 46). These manifest as enrichments across multiple pathways along with distinct variations in metabolites (notably involving amino acid and lipid metabolism disorders) (45, 46). This indicates that metabolic reprogramming drives the immune response from an initial pro-inflammatory phase to later immunosuppression, while being intricately regulated by underlying immune dysfunctions.

## 3 Key pathophysiological mechanisms of sepsis-associated CNS injury

**Figure 2:**
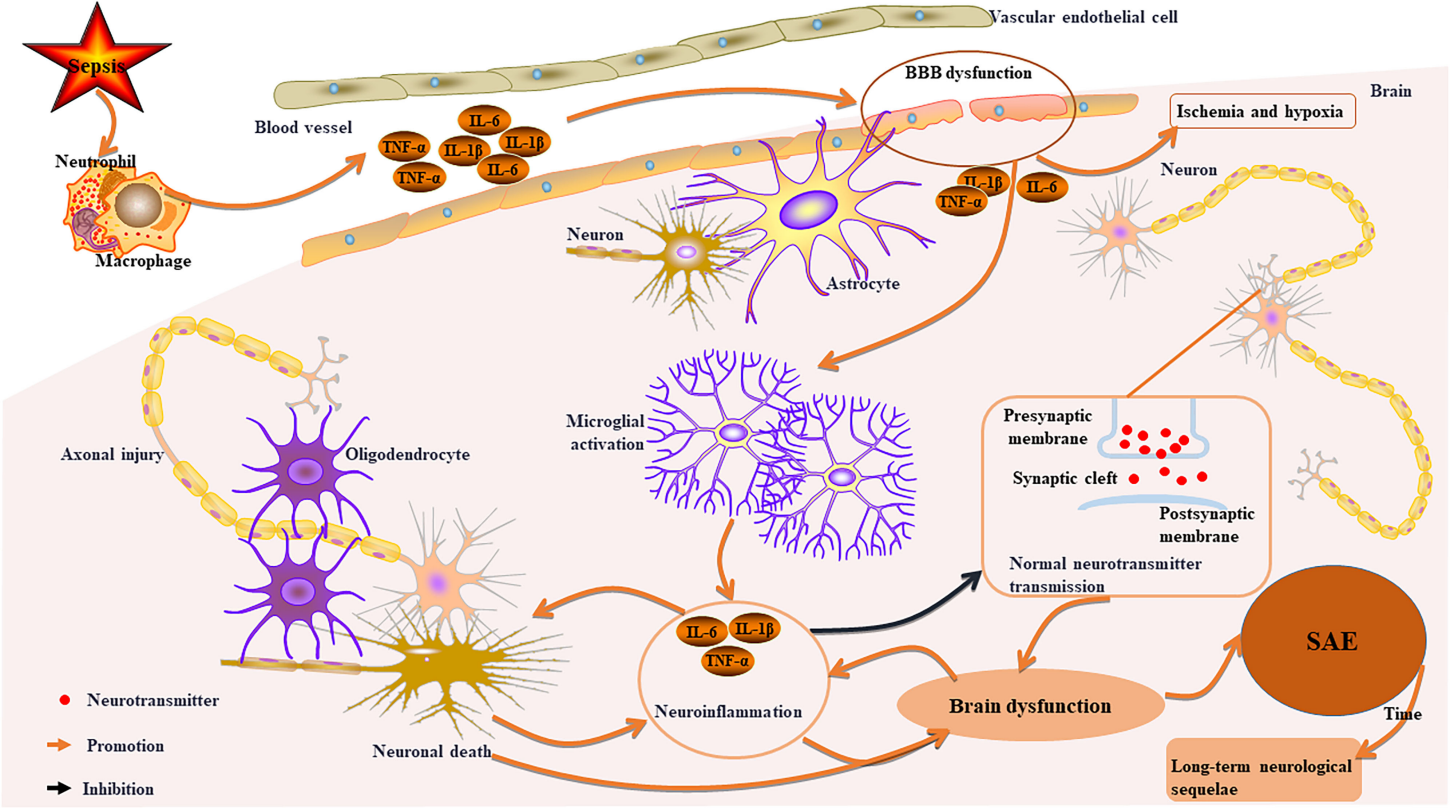
Key pathophysiological mechanisms of sepsis-related CNS injury. Sepsis activates immune cells, leading to systemic inflammation and damage to the BBB. This disruption allows inflammation into the brain, causing cerebral ischemia and hypoxia. Activated microglia release pro-inflammatory factors, resulting in neuroinflammation, axonal damage, neuronal death, and impaired neurotransmitter transmission, which contributes to brain dysfunction and SAE. Over time, survivors may experience long-term neurological sequelae. Furthermore, neuronal death can worsen neuroinflammation and brain dysfunction in a reciprocal manner.

SAE represents the primary clinical manifestation of CNS injury in patients suffering from sepsis (47–49). The pathophysiological underpinnings of SAE encompass four critical components (**Figure 2**): neuronal injury (50), aberrant activation of microglia (51), neuroinflammation and dysfunction of the blood-brain barrier (BBB) (52). Activated microglia initiate neuroinflammatory cascades and oxidative stress, leading to neuronal damage (48, 53). Importantly, this neuroimmune activation extends beyond the confines of brain tissue; it can also have systemic effects by releasing pro-inflammatory factors (such as TNF-α and IL-1β), thereby contributing to the onset and progression of dysfunction in peripheral organs (2, 48, 51, 52).

### 3.1 BBB dysfunction and neuroinflammation

The systemic inflammatory response initiated by sepsis serves as the primary catalyst for SAE (54). This pathological process results in significant damage to both the structure and function of the BBB, characterized by a down-regulation of tight junction proteins and an abnormal increase in permeability (55, 56). Such alterations facilitate the translocation of peripheral inflammatory mediators across the BBB into the central nervous system, ultimately disrupting neural microenvironment homeostasis (55, 56). Once the integrity of the BBB is compromised, microglia become markedly activated and adopt a pro-inflammatory phenotype (48).These activated microglia may cause damage to the structural integrity of neurons, impair synaptic plasticity, and disrupt neurotransmitter transmission by releasing pro-inflammatory factors (49, 57).

### 3.2 Abnormal cerebral perfusion and ischemic injury

Sepsis-induced BBB dysfunction and impaired cerebral autoregulation lead to cerebral hypoperfusion (58). The inflammatory response from sepsis facilitates the abnormal transfer of peripheral inflammatory molecules to the CNS, causing pathological accumulation of amyloid-β (Aβ) and tau proteins, which are linked to neurodegenerative diseases (59, 60). Clinical studies indicate that this accumulation in SAE patients not only worsens neurodegenerative conditions but is also associated with cerebrovascular diseases (59). Pathological evidence shows characteristic edema changes, ischemic injury, and infarction foci in the brain tissue of SAE patients, all closely related to microcirculation disorders (61).

### 3.3 Axonal injury and neuronal death

Sepsis can lead to axonal injury and neuronal death (62). In sepsis mouse models, axonal damage has been noted particularly in the cerebral cortex, thalamus, and hippocampus (57). This occurs due to a significant release of pro-inflammatory cytokines from the systemic inflammatory response triggered by sepsis (48). These cytokines activate microglia, which then release toxic mediators that harm neurons and axons directly (48). Furthermore, activated microglia may promote synaptic pruning, resulting in synaptic loss and neuronal death, which further exacerbates inflammation (63). Such changes may be causally linked to long-term cognitive dysfunction in patients with SAE (57).

### 3.4 Mechanism of long-term neurological sequelae

Survivors of SAE frequently experience what is known as “post-sepsis syndrome,” a condition characterized by persistent cognitive deficits, abnormal emotional regulation, and functional disabilities, among other symptoms (64, 65). These phenomena are associated with disrupted neural circuits and sustained neuroinflammation, which propagate systemic effects through the neuroimmune axis, ultimately impacting the functionality of various organ systems and perpetuating a detrimental cycle (65, 66).

## 4 CNS as a core driver of organ damage in sepsis

**Figure 3:**
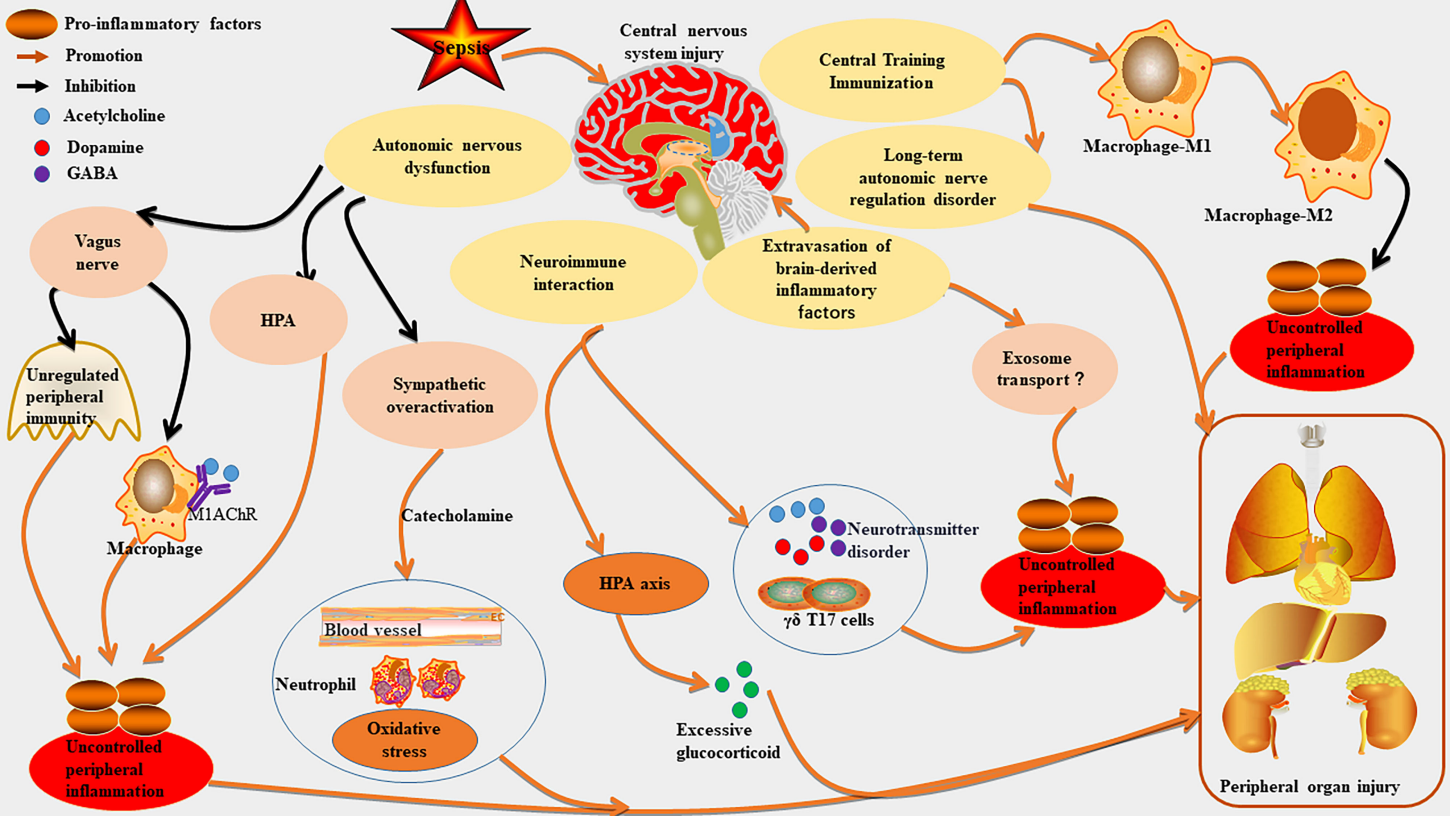
The mechanism of CNS as a core driver of organ damage in sepsis. Sepsis can worsen central nervous system (CNS) injury, which in turn exacerbates peripheral inflammation and organ damage. CNS injury may overactivated the sympathetic nervous system by disrupting autonomic nerve function, leading to excessive catecholamine release and impairing the vagal anti-cholinergic pathway, thus worsening peripheral inflammation. It also disrupts the neuroimmune regulatory network, hyperactivates the hypothalamic-pituitary-adrenal (HPA) axis, causes neurotransmitter transmission disorders, and promotes immune cell migration, further impacting peripheral immunity. Additionally, CNS injury can enhance peripheral inflammation by regulating central trained immunity and causing immune homeostasis imbalance. It may aggravate inflammation through the extravasation of brain-derived inflammatory factors and their transport via exosomes. Long-term CNS injury can lead to neurological sequelae and further intensify peripheral organ damage.

In sepsis, the CNS regulates peripheral immune responses and organ functions through complex interactions within the neuro-immune-endocrine network (8, 67). The mechanisms can be summarized into the below core pathways (**Figure 3**, **Table 1**).

**Table 1:** The key experimental findings supporting CNS-mediated regulation of peripheral organs.

Modeling method	Animal	Intervention	Outcome	Reference
CLP	Mouse	Inhibit the apoptosis of cholinergic neurons	Reduce lung injury	(9)
LPS	Mouse	Specifically activate α7nAChR (GTS-21)	Reduce kidney damage	(74)
LPS	Mouse	Specifically activate α7nAChR(GTS-21)	Reduce myocardial injury	(75)
CLP	Mouse	Specifically activate α7nAChR(GTS-21)	Reduce liver damage	(76)
CLP	Rat	Inhibit sympathetic activation	Reduce myocardial injury	(79)
Microbial peritonitis	Rat	Promote sympathetic activation	Impair respiratory function	(8)
LPS	Rat	Inhibit sympathetic activation	Reduce myocardial injury	(81)
CLP	Mouse	Inhibit necroptosis of GABAergic neurons	Reduce myocardial injury	(80)
CLP	Mouse	β-glucan-activated training immunity	Reduce liver and lung damage	(103)

### 4.1 Autonomic nerve dysfunction

#### 4.1.1 Damage to the vagus nerve cholinergic anti-inflammatory pathway

In animal models of sepsis, stimulation of the vagus nerve has been demonstrated to inhibit the synthesis of inflammatory cytokines, the recruitment of white blood cells, and the activation of endothelial cells (68, 69). The efferent fibers of the vagus nerve release acetylcholine, which interacts with nicotinic receptors on macrophages’ surface to suppress the production of pro-inflammatory cytokines (70, 71). Sepsis can induce inflammation in the brainstem, leading to damage in cholinergic anti-inflammatory pathway (CAP) function. This results in diminished efferent signals from the vagus nerve and a compromised ability to effectively mitigate peripheral inflammatory responses (9). Dysfunction within CAP contributes to an excessive release of pro-inflammatory factors, thereby exacerbating inflammatory damage to vital organs including the heart, lungs, and liver (72). Furthermore, downregulation of central cholinergic signaling mediated by M1 muscarinic acetylcholine receptors (M1AChR) may intensify peripheral inflammation (73–76). Experimental evidence also indicates that septic mice exhibit impaired functionality in vagus nerve preganglionic neurons located in the brainstem; this impairment weakens their capacity to regulate immune responses in peripheral organs such as the spleen (9).

#### 4.1.2 Excessive sympathetic nerve activation

Peripheral infection activates the brainstem’s sympathetic nerve via humoral or neural pathways, resulting in increased sympathetic output (8). In an Escherichia coli sepsis model, brainstem inflammation leads to sustained sympathetic hyperactivity and a massive release of catecholamines (8). This hyperactivity directly harms the myocardium (e.g., causing arrhythmias) (77) and worsens renal ischemic injury through vasoconstriction (8). Inhibition of the sympathetic nerve and the suppression of catecholamine release can mitigate myocardial dysfunction associated with sepsis (78–81). Catecholamines also promote neutrophil infiltration and oxidative stress, further aggravating lung (82) and liver injuries (83).

### 4.2 The neuro-immune regulatory network

The CNS not only responds to peripheral inflammation in sepsis but also regulates immune cell migration and inflammatory factor production, creating a “brain-peripheral organ” feedback loop (6). Sepsis can induce intestinal immune cells (e.g., IL-7R CD8 γδ T17 cells) to migrate to the CNS, altering local immunity and amplifying systemic inflammation, which exacerbates oxidative stress and tissue damage in organs like the liver, lungs, and heart (84). In addition, sepsis-induced neurotransmitter disorders can contribute to peripheral organ damage by influencing immune metabolism. For example, dopamine imbalance can affect immune metabolism via the dopamine-tyrosine decarboxylase 1 axis, resulting in inflammation-related immunosuppression during sepsis (85). Central neurotransmitters (like acetylcholine and γ-aminobutyric acid (GABA)) and cytokines (such as IL-1β) regulate peripheral immunity bidirectionally (86). Acetylcholine released from the vagal nucleus inhibits the inflammatory response of splenic macrophages; reduced acetylcholine transmission in the hippocampus during sepsis weakens this immunoinhibition (86). GABA influences macrophage maturation and inflammatory responses through GABA transporters (GAT2), with GAT2 deficiency leading to decreased IL-1β production in pro-inflammatory macrophages, thus alleviating inflammation (87). The activation of α2A adrenergic receptors in spinal astrocytes mitigates sepsis-induced cardiac injury by inhibiting the necroptosis of GABAergic neurons (80).Notably, uncontrolled peripheral immunity due to neurotransmitter disorders may worsen damage chains like the brain-liver and brain-gut axes (88, 89). The hypothalamic-pituitary-adrenal (HPA) axis is a key part of the neuroendocrine system that regulates peripheral immune responses through hormones such as glucocorticoids (e.g., cortisol), primarily exerting anti-inflammatory effects (90). HPA axis activation commonly occurs in acute sepsis phases, characterized by significantly elevated circulating cortisol levels (91, 92). Persistent hyperactivity of the HPA axis can disrupt negative feedback mechanisms (e.g., α1-adrenergic receptor desensitization), leading to excessive glucocorticoid exposure and worsening multi-organ damage (93, 94). This imbalance in communication may lead to persistent organ dysfunction (5, 95, 96). The aforementioned evidence suggests that CNS damage in sepsis plays a crucial role in the associated peripheral organ dysfunction by influencing immune cell migration, neurotransmitter transmission, and the HPA axis. However, the potential interactions among these factors require further investigation.

### 4.3 The regulatory role of central training immunity

Central training immunity refers to the immune memory established by progenitor cells in the bone marrow through metabolic and epigenetic reprogramming (e.g., histone modification) in response to specific stimuli (e.g., microorganisms), which enhances reactions to subsequent stimuli (97). Research indicates that sepsis can create persistent innate immune memory in the mouse brain, termed “trained innate immunity,” independent of peripheral immune systems (98). Specifically, microglia in the hippocampus of sepsis-surviving mice undergo epigenetic changes, entering a long-term high-responsiveness state (like pro-inflammatory M1 polarization) that increases vulnerability to neurotoxins and causes cognitive dysfunction and neural damage (98). Central trained immunity enhances the body’s defense against sepsis by boosting bone marrow progenitor cells’ response to pathogens (99). However, abnormal immune responses can lead to excessive inflammation and autoimmune damage (99). For example, immune memory in the brain may increase vulnerability to neurodegenerative diseases, contributing to cognitive dysfunction in sepsis survivors (98). Notably, specific stimuli like β-glucan can induce “trained immunity” in central immune cells, allowing for a stronger anti-inflammatory response upon secondary stimulation (100–102). Research indicates that central trained immunity may reduce organ damage from sepsis—such as in the liver and lung (103)—by regulating peripheral monocyte/macrophage phenotypic transformation (e.g., promoting conversion from pro-inflammatory M1 to anti-inflammatory M2) (100, 101, 104). The above explanation indicates that central training immunity has a dual role in sepsis: it enhances immune memory for protection while potentially increasing long-term vulnerability of the nervous and immune systems post-sepsis.

### 4.4 Brain-derived inflammatory factor extravasation

Sepsis can disrupt the BBB, resulting in an elevation of inflammatory factors within the brain, such as interleukin-6 (IL-6) and high mobility group box 1 (HMGB1) (48, 54). Radioactive labeling experiments have demonstrated that HMGB1 is capable of bidirectional translocation across the BBB—moving from blood to brain tissue and vice versa. Moreover, inflammation induced by lipopolysaccharides can accelerate its transport rate into peripheral circulation (105). Additionally, research indicates a significant increase in IL-6 expression within the brains of septic mice, with BBB dysfunction facilitating cytokine infiltration from brain tissue into peripheral regions (106). Furthermore, factors derived from the brain may indirectly influence vascular endothelial cell injury and contribute to damage in organs such as the lungs and kidneys through mechanisms related to neuroinflammation (47, 54, 107). It has also been established that exosomes containing inflammatory factors are capable of traversing the BBB and entering cerebral tissues to promote neuronal damage (108, 109). However, whether inflammatory factors produced in the brain can be transmitted via exosomes to affect peripheral organ integrity remains an area requiring further investigation. In summary, these findings suggest that sepsis compromises the integrity of the BBB through various mechanisms including HMGB1 nuclear translocation, receptor activation, and immune cell infiltration; this leads to leakage of inflammatory mediators like HMGB1. This process may involve structural damage that establishes a positive feedback loop for “brain-peripheral” inflammatory dissemination; however, additional studies are necessary for a comprehensive understanding of these underlying mechanisms.

### 4.5 Others

In addition, the SAE resulting from nervous system injury not only causes acute cognitive impairment but also has long-term effects on autonomic nerve regulation of organs. This is closely linked to “post-sepsis syndrome,” characterized by abnormalities in cardiovascular and renal functions observed in survivors (110, 111).

## 5 Intervention strategies targeting the core role of the CNS

Based on the core role of the CNS, current treatment strategies mainly focus on regulating CNS inflammation and immune imbalance (112, 113). First, direct neuroinflammation inhibition: 1) Selective elimination of reactive oxygen species, such as hydroxyl radicals, significantly reduces oxidative stress and neuroinflammation in the CNS, improving cognitive dysfunction related to sepsis (114); 2)Ferroptosis inhibitors like Liproxstatin-1 reduce neuronal lipid peroxidation while enhancing SAE outcomes and multi-organ function (1, 115); 3)Regulation of TLR4 signaling involves targeted inhibition of the microglial TLR4/NF-κB pathway to prevent neuroinflammation from spreading peripherally (116). Second, regulation of the neuro-immune axis: 1) Adenosine-lidocaine-magnesium therapy balances autonomic nerve activity, alleviating hyperactivity in sympathetic nerves and reducing organ inflammation (113); 2) Enhancing cholinergic pathways with α7nAChR agonists restores vagus nerve anti-inflammatory functions (9, 72). Third, BBB protection and repair: Glycoprotein modulation and APOH protein application improve BBB integrity while decreasing neuroinflammation and systemic inflammation (23, 117).; Vitamin C therapy offers antioxidant benefits that protect BBB structure and limit neuroinflammatory overflow (118). However, these intervention strategies remain in research stages requiring further clinical translation (119). Further optimization is essential. Current research indicates a discrepancy between the outcomes of animal experiments and human clinical trials regarding sepsis treatment drugs (120). Considering the pivotal role of the central nervous system in the pathophysiological processes associated with sepsis, future drug development should explore the integration of neuroprotective strategies alongside systemic anti-inflammatory treatments. This approach holds promise for disrupting the detrimental cycle characteristic of sepsis.

### 5.1 Limitations and challenges of current research

Although existing evidence suggests that a comprehensive treatment strategy centered on the CNS may pave the way for new clinical intervention pathways for patients with sepsis, several key scientific issues remain unresolved. First, the pathogenesis of sepsis is complex, and patients exhibit highly heterogeneous clinical manifestations. Current animal models still fail to fully replicate the disease process observed in humans. While some studies have compared cellular changes in brain tissue samples from patients with SAE and murine models, revealing certain similarities in immune microenvironment signaling (121), there is also evidence indicating that humans, mice, and other species display fundamental differences in blood immune characteristics due to varying sensitivities to inflammation (122). Furthermore, during bacteremia, significant disparities exist between humans and mouse models regarding bacterial clearance ability, phagocytic function, and cytokine induction levels in the bloodstream (123). These differences limit the reliability of current animal models in simulating human neuroimmune responses and consequently constrain the clinical translational value of related research and drug development. Second, current research predominantly relies on single-organ studies conducted within animal models; systematic multi-organ comparative analyses are lacking. Moreover, data derived from human tissue samples remains scarce. Third, the pathogenesis and intervention strategies for systemic inflammatory response syndrome (SIRS) differ between the acute and chronic phases. Currently, the specific mechanisms and dynamic changes regarding neuroimmune regulation in peripheral organ damage at various stages remain poorly understood. Fourth, the regulatory interactions among multiple organs present a highly complex network. In sepsis, the central nervous system modulates peripheral organ function through several mechanisms; concurrently, intestinal microbiota can influence brain inflammatory responses via the gut-brain axis. The extent to which other organs—such as the lungs, liver, and kidneys—may exacerbate central nervous system injury through analogous feedback loops warrants further investigation. Fifth, conclusive evidence demonstrating that brain-derived inflammatory factors directly induce peripheral organ damage is still lacking. Additionally, whether these brain inflammatory factors contribute to further harm in other organs through carriers such as exosomes needs to be thoroughly examined. Sixth, the dynamic regulatory mechanisms of the neuro-immune-organ axis are not yet fully elucidated. For instance, it remains unclear how specific interactions between neuroendocrine factors (including HPA axis hormones and catecholamines) and the immune system mediate peripheral organ injury.

Given the aforementioned research limitations and challenges, future investigations should prioritize the development of sepsis models that more closely mimic the characteristics of human disease, such as organoids or organ-on-a-chip systems, to effectively simulate the neuro-immune-organ regulatory network. Furthermore, it is essential to integrate multi-modal data—including clinical parameters, imaging information, and biomarkers—to construct predictive models for sepsis neuro-immune regulation, thereby facilitating individualized treatment approaches. Moreover, adopting a longitudinal research strategy that combines single-cell sequencing with spatial transcriptomics techniques is recommended to systematically illustrate the dynamic evolution of the neuro-immune-organ network throughout the disease course and analyze its regulatory patterns. The utilization of multi-organ chip or organoid co-culture systems to emulate neuro-organ interactions under septic conditions can also provide valuable insights into molecular mechanisms at play.

### 5.2 Prospects

Overall, the CNS plays a crucial role in the pathogenesis and prognosis of sepsis, acting as both an early target for damage and a key regulator driving multi-organ dysfunction through neuroinflammation, neuroimmune dysregulation, and autonomic dysfunction. A comprehensive understanding of the CNS’s fundamental role in sepsis is essential for elucidating its underlying pathophysiology and provides a theoretical foundation for developing an integrative treatment strategy focused on neuroimmune regulation. Ultimately, this approach aims to enhance clinical outcomes for patients suffering from sepsis.
